# Neuroprotection by Therapeutic Hypothermia

**DOI:** 10.3389/fnins.2019.00586

**Published:** 2019-06-11

**Authors:** Ying-Jian Sun, Zi-Yuan Zhang, Bin Fan, Guang-Yu Li

**Affiliations:** Department of Ophthalmology, The Second Hospital of Jilin University, Changchun, China

**Keywords:** hypothermia, neuroprotection, cold-induced protein, neurological diseases, stroke

## Abstract

Hypothermia therapy is an old and important method of neuroprotection. Until now, many neurological diseases such as stroke, traumatic brain injury, intracranial pressure elevation, subarachnoid hemorrhage, spinal cord injury, hepatic encephalopathy, and neonatal peripartum encephalopathy have proven to be suppressed by therapeutic hypothermia. Beneficial effects of therapeutic hypothermia have also been discovered, and progress has been made toward improving the benefits of therapeutic hypothermia further through combination with other neuroprotective treatments and by probing the mechanism of hypothermia neuroprotection. In this review, we compare different hypothermia induction methods and provide a summarized account of the synergistic effect of hypothermia therapy with other neuroprotective treatments, along with an overview of hypothermia neuroprotection mechanisms and cold/hypothermia-induced proteins.

## Introduction

A number of experimental and clinical studies have provided evidence in support of the neuroprotective effects of induction of hypothermia. The earliest recording of hypothermia as a therapeutic agent is dated more than 5000 years old, coming from an ancient Egyptian Edwin Smith Papyrus (Wang et al., 2006). In ancient times, hypothermia therapy consisting of ice packs was used to treat hemorrhage, and said therapy was also widely used in cardiac arrest (Dzieciol et al., 2014), comatose patients (Dell’Anna et al., 2014) and other diseases. There was an apparent interest in the exploration of the mechanism(s) of hypothermia neuroprotection. A role of hypothermia has now been reported in many neurological diseases, for instance, stroke, traumatic brain injury, intracranial pressure elevation, subarachnoid hemorrhage, spinal cord injury, hepatic encephalopathy, and neonatal peripartum encephalopathy (Karnatovskaia et al., 2014). However, it has also been reported that hypothermia may not be neuroprotective (Clifton et al., 2001; Hutchison et al., 2008; Maekawa et al., 2015). Such discrepancies in literature might perhaps be related to the duration of cooling time and the methods used to induce hypothermia (Clifton et al., 2001; Hutchison et al., 2008; Wowk et al., 2014; Maekawa et al., 2015). The methods employed to induce hypothermia have profound effect on the resulting neuroprotection. Combination with other treatment methods has been explored as a means to enhance the benefits of hypothermia protection. Even after several reports on the topic, the mechanisms, by which hypothermia affords neuroprotection, remain unclear. It is believed that hypothermia-induced neuroprotection might be due to decreased metabolism, reduced generation of radicals, ameliorated inflammation and inhibition of excitotoxicity and apoptosis. Further, the importance of cold-induced proteins as important components of hypothermia neuroprotection has also been realized. In this review, we have summarized methods of hypothermia induction, and the effectiveness of combination of other neuroprotective methods with hypothermia and the cold-induced proteins. We hope that this article will provide guidance for future pre-clinical studies and the clinical trials on hypothermia neuroprotection.

## Hypothermia Induction Methods

Experimental as well as clinical data points to a proven neuroprotective effect of therapeutic hypothermia. Also, hypothermia induction methods have an influence on the hypothermia effect. The two most commonly used methods for induction of hypothermia are local hypothermia and general hypothermia.

Local hypothermia provides precise hypothermic regions in the damaged area and the rectal temperature is kept 34–35°C to minimize the potential side effects of hypothermia. Many physical methods are used to achieve local hypothermia, for example, a cooling helmet is a good way to achieve rapid and selective brain hypothermia for a stroke or head injury patient (Wang et al., 2004; Ikeda et al., 2012). In a research study comprising of 15 patients following resuscitation, selective head cooling by a cooling helmet decreased urinary 8-OHdG levels on days 6 and 7 (Ikeda et al., 2012). Bennet et al. (2007) used a cooling coil made from silicone tubing in a severe hypoxia model of preterm fetal sheep for local hypothermia and observed reduced loss of neurons and immature oligodendroglia. In a study on spinal cord injury research (Bazley et al., 2014), the system included a heat exchanger constructed from copper tubing, bent into four layers that were all equal in length, measuring 4.4 × 0.8 inches each, and the tubing was inserted under the skin over the paravertebral muscle extending from the T6 to T10 spinal segments. By circulating cold water, local hypothermia was achieved and was found to be beneficial for spinal cord injury.

**FIGURE 1:**
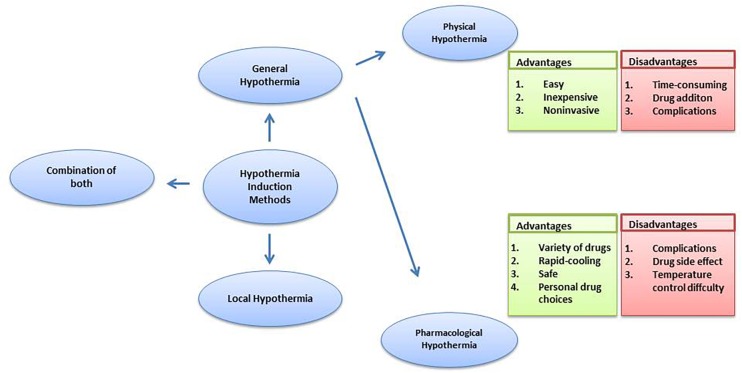
The comparison of hypothermia induction methods.

In general therapy, a 33–34°C rectal temperature is maintained, creating a moderate systemic hypothermia (Shankaran et al., 2005; Azzopardi et al., 2008; Jacobs et al., 2011). Further, for general hypothermia, two approaches commonly used are physical hypothermia and pharmacological hypothermia. Reducing ambient temperature, using a cooling blanket or ice pad and infusing rapidly cooled saline are considered physical hypothermia (Ikeda et al., 2012). Pharmacological hypothermia is related to drugs such as the neurotensin (NT) (Zhang et al., 2013). Gu et al. (2015) revealed the potential therapeutic effects on stroke and traumatic brain injury of adult rodents of Neurotensin receptor-1 (NTR1) agonist HPI201 (formerly known as ABS201)-induced hypothermia. HPI201-induced hypothermia resulted in markedly reduced MMP-9 levels and caspase-3 activation. NTR1 agonist induced hypothermia via the NTR receptor in the brain (Dubuc et al., 1999). Hwan et al. (2014) demonstrated that HPI-363 is approximately 10 times more potent than HPI-201 in inducing therapeutic hypothermia. HPI-363 is the analog of NT (8–13). This is because of the C-terminal hexapeptide that has structural elements critical for complete biological activity (Carraway and Leeman, 1975). The biologically stable NT (8–13) analogs can penetrate the blood–brain barrier (Kokko et al., 2005), while original NTR1 agonists cannot. Anesthetic is another method used to induce hypothermia (Whittington et al., 2013). It has been reported that isoflurane-induced hypothermia attenuates the early phase blood-brain barrier disruption in cerebral ischemia (Liu et al., 2017). N-cyclohexyladenosine (CHA), an A_1_ adenosine receptor (A_1_AR) agonist, also induced hypothermia, and animals subjected to cardiac arrest and cooled by CHA survived better and exhibited less neuronal cell death (Jinka et al., 2015). Further, the agonist of transient receptor potential vanilloid channel 1 (TRPV1), dihydrocapsaicin (DHC), is used in pharmacological hypothermia as well (Zhang J. et al., 2018). Compared with pharmacological cooling processes, physical cooling processes are costly and time-consuming (Alexander et al., 2012). It may be because of shivering, a defensive metabolic response to cold, that works against temperature reduction. Therefore, anesthesia has to be used in patients to combat the cold defense response, which has the potential to lead to infection and possibly other side effects due to prolonged hypothermia (Schwab et al., 1998), while NTR compounds lead to a lack of shivering (Gu et al., 2015). Also, physical cooling has associated complications such as hypotension, arrhythmia and change of fluid pH (Gröger et al., 2013; Mohr et al., 2013; Stuart et al., 2013). It seems that pharmacological reagent-induced controlled hypothermia, which targets the brain thermoregulatory center, has emerged as an efficient and considerably safer treatment for patients, with a further benefit being that a lot of choices of drug are provided. Zhang et al. (2013) reviewed the neuroprotective effects of eight classes of hypothermia-inducing drugs: the cannabinoids, opioid receptor activators, transient receptor potential vanilloid, neurotensins, thyroxine derivatives, dopamine receptor activators, hypothermia-inducing gasses, adenosine and adenine nucleotides. However, pharmacological intervention still has its limitations. Just like NT (8–13) analogs, the drugs caused severe hypothermia (<30 degrees) leading to the requirement for more significant re-warming measures (Tyler-McMahon et al., 2000a,b; Katz et al., 2004; Smith et al., 2011). Drugs also have the associated problem of drug resistance and hypothermic tolerance. Every drug application may disturb the balance of whole body such as the metabolic and cardiovascular systems (Piehl et al., 2011), in addition to the face that each person has individual differences in the drug dose they require. Moreover, it has been demonstrated that the combination of low DHC and ice pads significantly improves every measured outcome, compared to low DHC or the ice pad alone. Combination therapy achieved hypothermia faster; reduced more neurological deficits and decreased apoptotic cell death (Zhang J. et al., 2018). Barks et al. (2010) found that the combination therapy sustained more benefits in late outcome assessment of cerebral hypoxia-ischemia. These observations clearly indicate that combining physical and pharmacological hypothermia could be a promising therapy (Figure 1).

In addition, some comparisons were made between general hypothermia and local hypothermia. In a global ischemia research study, a water-cooling blanket (the rapid infusion of cooled saline, gastric lavage with cooled saline) and sedation were used to cool the whole body. Head cooling was achieved by a cooling helmet. These two neuroprotections are similar, and whole-body cooling had a greater effect on the suppression of radical production than head cooling (Ikeda et al., 2012). A research study comparing selective head cooling therapy with whole body cooling therapy in newborns with hypoxic ischemic encephalopathy uncovered no difference between the two methods in terms of adverse effects and short-term results (Atici et al., 2015). Other research studies have also reached similar conclusion about hypoxic ischemic encephalopathy (Sarkar et al., 2009a,b; Celik et al., 2016). However, it was reported that local hypothermia is better than general hypothermia with a significantly lower rate of severe cortex lesions (Rutherford et al., 2005). It has been reported that local hypothermia may be more suitable for longer durations of hypothermia treatment for spinal cord injury because it does not require as much temperature change in healthy tissues (Bazley et al., 2014). In conclusion, local hypothermia may be the best option for providing similar protection to general hypothermia and reducing temperature effects throughout the body, along with minimizing side effects. Further studies are still necessary to compare the adverse effects between local and general hypothermia and reach a definitive conclusion.

## Hypothermia Combined With Other Neuroprotective Methods

In addition to hypothermia neuroprotection, there are other treatments that are applied to nerve injury. However, the combination of hypothermia and other treatments was found to produce a greater neuroprotective effect. These therapies are divided into three categories: cell therapy (Table 1), drug therapy (Table 2), and other therapies (Table 3).

**Table 1:** The combination of hypothermia and cell therapy.

Combination strategy	Model	Diseases	References
Neural stem cells transplantation	Spinal cord injury rat model	Spinal cord injury	Zhu et al., 2015
Cell-scaffold complex seeded with Nogo receptor (NgR)-silenced neural stem cells and Schwann cells transplantation	Spinal cord injury rat model	Spinal cord injury	Wang D. et al., 2014
Neural stem cells transplantation	Carotid artery ligation rat model	Hypoxic-ischemic encephalopathy	Wang L. et al., 2014
Mesenchymal stem cells transplantation	Carotid artery ligation rat model	Hypoxic-ischemic encephalopathy	Park et al., 2015
Mesenchymal stem cells transplantation	Lateral fluid percussion brain injury rat model	Traumatic brain injury	Tu et al., 2012
Adipose-derived stem cells transplantation	Middle cerebral artery occlusion (MCAO) rat model	Stroke	Zhao et al., 2018

**Table 2:** The combination of hypothermia and drug therapy.

Combination strategy	Model	Diseases	References
i.v. 1 mg/kg (0.2 m L/kg) of Cannabidiol	Carotid artery ligation rat model	Hypoxic-ischemic encephalopathy	Lafuente et al., 2016
i.p. 2.5 mg/kg of bumetanide	Carotid artery ligation rat model	Hypoxic-ischemic encephalopathy	Liu et al., 2012
i.p. 0.05 ml/10 gm of Docosahexaenoic Acid (DHA)	Carotid artery ligation rat model	Hypoxic-ischemic encephalopathy	Berman et al., 2013
i.v. 0.0016 PNAU/100 g human urinary kallidinogenase	Carotid artery ligation rat model	Hypoxic-ischemic encephalopathy	Gao et al., 2018
i.p. 40 mg/kg of phenobarbital	Carotid artery ligation rat model	Hypoxic-ischemic encephalopathy	Barks et al., 2010
p.o. 0.5 mg/g exendin-4	Carotid artery ligation mouse model	Hypoxic-ischemic encephalopathy	Rocha-Ferreira et al., 2018
i.p. 20 mg/kg of topiramate or memantine/ 0.01–1 μM topiramate and 1–30 μM memantine	Carotid artery ligation rat model/OGD organotypic hippocampal slice model	Hypoxic-ischemic encephalopathy	Rocha-Ferreira et al., 2018
i.v. 200 IU/kg of erythropoietin	Clinical patients	Hypoxic-ischemic encephalopathy	Lv et al., 2017
s.c. 15 mg/kg of G-CSF	MCAO rat model	Stroke	Ghahari et al., 2014
p.o. 1 mg/kg of atorvastatin	MCAO rat model	Stroke	Lee et al., 2008
i.p. 10 mg/kg of HPI 201	MCAO rat model	Stroke	Lee et al., 2016
i.v. 1 mg/kg of chlorpromazine and 1 mg/kg of promethazine	MCAO rat model	Stroke	Liu S. et al., 2015
i.v. 300 mg/kg of valproic acid	Cardiac arrest rat model	Cardiac arrest	Oh et al., 2017
i.v. 4 ml/kg of Emulsified isoflurane (EIso)	Cardiac arrest rat model	Cardiac arrest	Wu et al., 2017
i.v. 3 mg/kg of tacrolimus	Lateral fluid percussion brain injury rat model	Traumatic brain injury	Oda et al., 2011
1 mmol/L VPA	Cobalt chloride (Co Cl_2_) induced -hypoxia cell model	Cerebral ischemic and traumatic brain injury.	Jin et al., 2014
i.p. 400 mg/kg of citicoline	MCAO rat model	Cerebral ischemic	Sahin et al., 2010
Infusion, 5 mg/kg of melatonin	Perinatal asphyxia piglet model	Hypoxic-ischemic encephalopathy	Powell et al., 2011; Robertson et al., 2013
Infusion,1 mg/kg HET0016	Asphyxia piglet model	Hypoxic-ischemic encephalopathy	Zhu et al., 2015
40 μM dantrolene	OGD/R cell model	Stroke	Xu et al., 2015
100 nM C5a RA	OGD/R cell model	Stroke	Thundyil et al., 2012

**Table 3:** The combination of hypothermia and other therapies.

Combination strategy	Model	Diseases	References
Xenon	Carotid artery ligation rat model/OGD cell model	Hypoxic-ischemic encephalopathy	Ma et al., 2005
Intravenous loading dose of 360 μmol/kg MgSO_4_ before ischemia followed by intravenous infusion (IVI) at 120 μmol/kg	2 vessel occlusion with hypotension rat model	Stroke	Zhu et al., 2005
Bloodletting at Jing points	Cortical contusion injury rat model	Traumatic brain injury	Tu et al., 2016

For cell therapy, stem cells are differentiated into a variety of cells within the nervous system in order to be used for the treatment of nerve diseases. Wang and coworkers found that combination treatment with therapeutic hypothermia produced synergistic effects in transplantation to promote the recovery of spinal cord injury (Wang D. et al., 2014; Zhu et al., 2015), while in hypoxic-ischemic encephalopathy, it was found to exert simple neuroprotective effects (Wang L. et al., 2014). On neonatal hypoxia-ischemic encephalopathy, mesenchymal stem cells transplantation combined treatment with hypothermia proved to be a better therapy than either therapy alone (Park et al., 2015). Furthermore, the temperature-sensitive mesenchymal stem cells from an umbilical cord, infected with a retrovirus carrying the temperature-sensitive A58 SV40 LT antigen gene, were applied to the traumatic brain injury. In this study, the greatest protective effect on the recovery of neurological function was the therapy which combined temperature-sensitive mesenchymal stem cells and hypothermia (Tu et al., 2012). Additionally, for treating stroke, adipose-derived stem cells combined with hypothermia produced a superior approach (Zhao et al., 2018). Moreover, many drugs enhanced therapeutic hypothermia neuroprotection in nerve injury. They included chemical drugs, hormones, neuroprotectants and others. For example, valproic acid is a histone deacetylase inhibitor. Jin et al. (2014) showed that the combined treatment with valproic acid and hypothermia improves survival and decreases cell death after chemically induced hypoxia in HT22 hippocampal cells. Valproic acid also enhanced neuroprotective effect of hypothermia against ethanol-mediated neuronal injury, and improved survival in a rat cardiac arrest model (Oh et al., 2017; Vishwakarma et al., 2017). Bumetanide, a clinically available loop diuretic, inhibited NKCC1 and improved the neuroprotective efficacy of treatment with phenobarbital and hypothermia in a neonatal cerebral hypoxia-ischemia model (Liu et al., 2012). Cannabidiol (CBD), the main non-psychoactive component of *Cannabis sativa*, has recently been shown to produce additive effect with hypothermia, resulting in a greater overall benefit in the early HI brain damage (Lafuente et al., 2016). Combined emulsified isoflurane and hypothermia treatment results in significant improvements in survival and neurological outcomes in a rat model of cardiac arrest (Wu et al., 2017). Dantrolene enhances the protective effect of hypothermia on OGH/R cerebral cortex neurons (Xu et al., 2015). Early post-hypoxia-ischemia administration of phenobarbital may augment the neuroprotective efficacy of therapeutic hypothermia (Barks et al., 2010) and some hormones have a neuroprotective effect in augmenting hypothermia protection. Melatonin augmented hypothermic neuroprotection in a piglet model of perinatal asphyxia (Powell et al., 2011). Exendin-4 is an analog of the human glucagon-like peptide-1 (GLP-1) gut hormone peptide. In a study by Rocha-Ferreira et al. (2018), exendin-4 was found to enhance the neuroprotection of therapeutic hypothermia. The combined therapy with human urinary kallidinogenase (HUK) and hypothermia enhanced the efficiency by promoting angiogenesis and regeneration and rescuing tight-junction loss in HIE rat model (Gao et al., 2018). And for erythropoietin, no significant benefit was observed from treatment with combination therapy in HIE rat experiments (Fang et al., 2013). However, erythropoietin combined with hypothermia reduced serum Tau protein levels and improved neonatal behavioral neurology outcomes but did not affect long-term neurodevelopmental outcomes in neonatal patients (Lv et al., 2017). Neuroprotectants, such as G-CSF, were used in brain injury. Hypothermic treatment plus G-CSF significantly reduced mortality rate and edema and improved neurological function in the rat transient middle cerebral artery occlusion (MCAO) model (Ghahari et al., 2014). Atorvastatin enhances hypothermia-induced neuroprotection after stroke (Lee et al., 2008). And in a study by Gao et al. (2014), a series of neuroprotectants including albumin, atorvastatin, baclofen, brain-derived neurotrophic factor, bumetanide, citicoline sodium salt hydrate, cyclosporine A etc., were applied to a oxygen-glucose deprivation and re-oxygenation-mediated neuronal injury. This research showed that combination of therapeutic hypothermia with brain derived neurotrophic factor, glibenclamide, dizocilpine, HUK or neuroglobin provided a better protection compared with a single treatment method. There are some other drugs like chlorpromazine, promethazine, citicoline and HET0016, which also augment therapeutic hypothermia protection (Sahin et al., 2010; Liu S. et al., 2015; Zhu et al., 2015). Furthermore, xenon, MgSO_4_ and Chinese traditional bloodletting treatment also offered better neuroprotection when combined with hypothermia (Ma et al., 2005; Zhu et al., 2005; Tu et al., 2016).

Most of these combined treatments are confirmed to be more effective than any other treatment being used alone. They can play their therapeutic role via many ways, such as scavenging free radicals, reducing energy consumption, reducing excitotoxicity and so on (Zhang Z. et al., 2018). However, there is no denying that some combined treatments did not exhibit a synergistic effect, such as that argon augmented therapeutic hypothermia which does not improve functional recovery in cardiac arrest, but may even worsen neurologic function. These findings suggest that future studies are warranted to investigate more specific mechanisms and modulating factors in neuroprotection.

## Mechanisms of Hypothermia-Mediated Protection

Therapeutic hypothermia is a promising neuroprotective intervention which has been shown to improve outcomes from nerve injury in humans. The neuroprotective role of hypothermia has been well established in experimental animals and in patients with cardiac arrest (Hakim et al., 2018), hypoxic-ischemic encephalopathy (Yum et al., 2018), traumatic brain injury (Leng, 2017) and other diseases (Zhu et al., 2015). Although the neuroprotective mechanisms of hypothermia in different diseases vary and have yet to be fully determined, the neuroprotection has been commonly ascribe to its effect on decreasing the metabolic rate, reducing the generation of radicals, ameliorating inflammation, inhibiting excitotoxicity and apoptosis.

Hypothermia decreases the metabolic rate of neurons after spinal cord injury, traumatic brain injury and other diseases. Metabolic changes associated with hypothermia include preserving glucose (Schaller and Graf, 2003), inhibited lactate generation (Drenger et al., 1997), increased plasma levels of glycerol (Wang et al., 2007), free fatty acids and ketoacids (Aoki et al., 1993). These metabolic changes induced by hypothermia are beneficial to preservation of pH and ATP of tissue and cell which promotes homeostasis (Kuffler, 2010).

The generation of free radicals and nitric oxide is considered to be associated with neuron damage (Lewen et al., 2000). Hypothermia, however, significantly inhibits superoxide and lipid peroxidation to decrease the generation of free radicals. Hypothermia was found to decrease the levels of ROS induced by ischemic stroke (Gao et al., 2014) and suppress the elevation in internal jugular NO after cerebral ischemia-reperfusion (Kumura et al., 1996).

Inflammation is involved in the occurrence and development of diseases, such as, cerebral ischemic injury. Further, hypothermia modulates inflammatory factors to reduce the inflammatory response. In acute brain injury, complement activation stimulates neutrophil pathways. Pro-inflammatory cytokines, including IL-1β, IL-6, IL-18, and TNF, are increased, which exacerbate neuronal injury (Huang et al., 2006; Nilupul Perera et al., 2006). Hypothermia has been shown to decrease the pro-inflammatory cytokines and increase anti-inflammatory cytokines production to inhibit inflammatory response (Vitkovic et al., 2001; Yatsiv et al., 2002; Hofstetter et al., 2007). However, anti-inflammatory cytokines such as IL-10 can also be reduced by hypothermia (Huet et al., 2007). It, therefore, appears that hypothermia may have a complex role in inflammatory modulation to protect neurons, that still needs to be elucidated.

Hypothermia also provides a neuroprotective benefit by decreasing excitotoxicity. The accumulation of excitotoxic amino acids, such as glutamate, is proven important in the pathogenesis of neuron damage (Dumont et al., 2001; Colbourne et al., 2003; Park et al., 2004; Sahuquillo and Vilalta, 2007; Mazzone and Nistri, 2011). It has been reported that hypothermia inhibits the release of glutamate in a rat spinal cord ischemia model (Ishikawa and Marsala, 1999). Globus et al. (1995) demonstrated that hypothermia reduces the extent of neuronal damage in traumatic brain injury by decreasing excessive extracellular release of glutamate and generation of hydroxyl radicals. Hypothermia reduced the release of glutamate by down-regulating the AMPA (α-amino-3-hydroxy-5-methy1-4-isoxazole-propinoic acid) to limit calcium influx and up-regulating the human glial glutamate transporter (hGLT-1).

**FIGURE 2:**
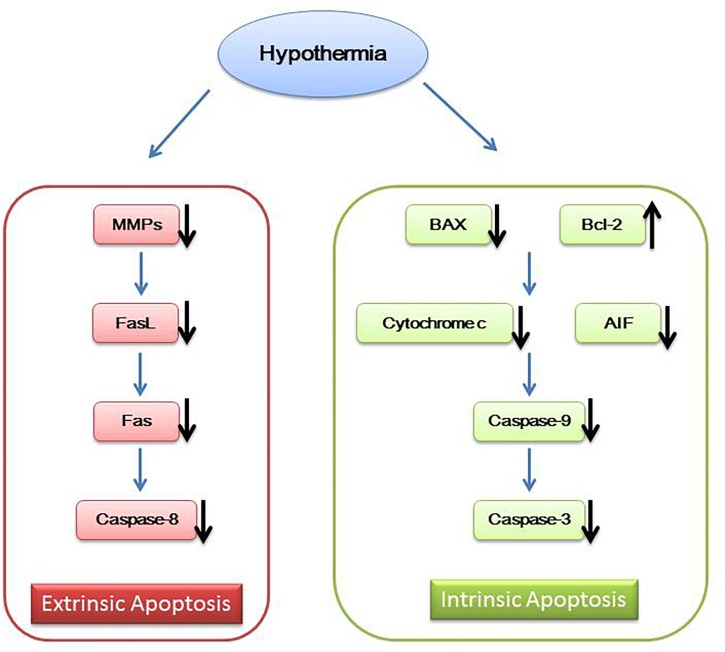
Hypothermia and apoptosis.

In addition to the above mechanisms, hypothermia also works by inhibiting neuron cell apoptosis. Mild hypothermia can interfere with the intrinsic and extrinsic cell apoptosis. Intrinsic cell apoptosis is associated with caspase family of apoptosis mediators. Neuron cell injury signals promote translocation of pro-apoptotic protein Bax and Bid from cytosol to the mitochondrial membrane changing the mitochondrial membrane potential and releasing cytochrome-c and apoptosis inducing factor (AIF) (Plesnila et al., 2001; Yenari et al., 2002; Shamas-Din et al., 2011). Then, cytochrome-c activates caspase-9 and caspase-3, leading to cell apoptosis (Zhu et al., 2004; Ohmura et al., 2005; Ok et al., 2012). Extrinsic cell apoptosis is mediated by Fas/FasL. Increased Fas activates caspase-8, resulting in cell apoptosis (Liu et al., 2008; Ehrenschwender and Wajant, 2009). Mild hypothermia increases Bcl-2, reduces cytochrome c release, inhibits the expression of BAX and decreases caspase family members such as caspase-9, caspase-8, and caspase-3 (Zhu et al., 2004; Ohmura et al., 2005; Ok et al., 2012; Sun et al., 2019). Mild hypothermia also inhibits the expression of matrix metalloproteinases (MMPs) to affect FasL (Lee et al., 2005), eventually leading to reduced Fas and caspase-8 (Liu et al., 2008; Figure 2).

## Cold Induced Protein

Although the key mechanisms have not been established clearly, cold induced proteins may play a key role in hypothermia neuroprotection, which suggests their targeting as novel therapeutic drug targets. Plenty of studies have uncovered a neuroprotective effect, focusing on these proteins. Cold-inducible RNA-binding protein (Cirbp) and cold-inducible RNA-binding protein motif 3 (RBM3) are the most widely studied proteins in this respect.

Cirbp, discovered in 1997, is a RNA-binding factor composed of a N-terminal RNA Recognition Motif (RRM) and a C-terminal region containing several repeats of the RGG motif (Zhu et al., 2016a). This protein is detected in low-level expression in human pancreas, heart, thyroid and other cells (Nishiyama et al., 1997). Xue et al. (1999) detected the expression of this protein in brains, lungs, stomachs and spinal cords of rats. When the temperature drops, expression levels of Cirbp are increased in PC12, K562, NC65 and other cell lines, and the expression of Cirbp in cells is decreased when the temperature is increased. Besides, in TNF-α-induced mouse fibroblast cells, hypothermia inhibited the apoptosis, and Cirbp levels were increased (Sakurai et al., 2006). Further, overexpression of Cirbp or application of recombinant Cirbp have shown protection against cell apoptosis (Liu J. L. et al., 2015; Wang and Zhang, 2015). In addition, knocking down Cirbp, with shRNA-Cirbp, blocked neuroprotection under hypothermic conditions in H_2_O_2_-induced apoptosis and caspase-dependent neuron apoptosis (Liu et al., 2012; Wang and Zhang, 2015). All these observations reveal that Cirbp is involved in the protection of tissues and organs by hypothermia. The RRM and repeats of RGG motif enhance binding of Cirbp to the 3′ UTR of TRX mRNA, resulting in enhanced expression of TRX in cells (Yokomizo et al., 1995). TRX is a well-known ROS scavenger (Park et al., 1999; Sahara et al., 2002), and Since ROS plays a crucial role in initiating caspase-dependent pathways through induction of TRX, Cirbp may suppress intrinsic cell death mainly through TRX. Furthermore, it has been demonstrated that caspase-dependent apoptosis protein Bax, caspase-3 and caspase-9 are inhibited, and anti-apoptotic protein Bcl-2 upregulated in the rat brain cortex neurons, through upregulation of Cirbp (Wang and Zhang, 2015). Xia et al. (2013) uncovered that Bcl-2 was upregulated and Cirbp was overexpressed in BALB/c mouse testicles. Also, cirbp inhibits DNA damage-induced apoptosis by downregulating caspase-3 (Lee et al., 2015). These studies indicate Cirbp to be a regulator of caspase-dependent apoptosis pathway, in which overexpression of Cirbp can downregulate caspase-3 and other apoptosis-related proteins, while knockdown of Cirbp exacerbates these proteins expression. In addition, the level of phosphorylated extracellular signal-regulated kinase1/2 (ERK1/2) upregulation was observed in H_2_O_2_ and TNF-α-induced cell death (Sakurai et al., 2006; Liu J. L. et al., 2015) and the protection of hypothermia was weakened when an ERK inhibitor was used (Sakurai et al., 2006). Moreover, increased NF-κB activity was observed with upregulating Cirbp, when mice were exposed to lower temperatures (Sakurai et al., 2006; Kaneko and Kibayashi, 2012). These data suggest that Cirbp protects cells from apoptosis partly through activating the NF-κB signaling. However, the mechanism of Cirbp in hypothermia protection is still unclear and needs more studies for a better understanding.

RBM3 is also a cold-induced protein which is induced by hypothermia (Derry et al., 1995; Danno et al., 1997) and has an impact on neuroprotection against various toxic insults such as hypoxia, UV and nitric oxide (Rosenthal et al., 2017; Yang et al., 2017; Zhuang et al., 2017). One study has further discussed whether mild hypothermia and RBM3 prevents neural cells from UV irradiation-elicited apoptosis using human neuroblastoma cell line SH-SY5Y as a cell model for neural cell death (Zhuang et al., 2017). It was indicated that mild hypothermia protected SH-SY5Y cells from UV irradiation-induced apoptosis. However, the protective effect of mild hypothermia was abrogated when RBM3 was silenced. On the contrast, SH-SY5Y cells could be rescued from UV-induced apoptosis when RBM3 was overexpressed. Obviously, RBM3 is the key mediator of mild hypothermia-related protection against UV in neuroblastoma cells. There is also a study evaluating whether RBM3 can inhibit staurosporine-induced apoptosis in neuron-like PC12 cells (Chip et al., 2011). Mild hypothermia profoundly promoted RBM3 expression and rescued neuronal cells from apoptosis. After blocking RBM3 expression in neuronal cells by specific siRNAs, the neuroprotective effect of hypothermia was significantly diminished, and RBM3 over-expression provided neuroprotection in the absence of hypothermia. Taken together, it is apparent that RBM3 is involved in hypothermia-induced neuroprotection. Furthermore, several studies have indicated that pro-apoptotic proteins Bax, Bad, apoptotic protein PARP and caspase-3 are downregulated when the expression of RBM3 is increased under hypothermia, while the anti-apoptotic protein Bcl-2 is induced (Chip et al., 2011; Ferry et al., 2011; Zhu et al., 2016b; Zhuang et al., 2017). Yang et al. (2017) found that RBM3 protects neuroblastoma cells from NO-induced apoptosis by suppressing p38 signaling, which mediates apoptosis through miR-143 induction. It has been reported that RBM3 is the key mediator of mild hypothermia-related protection against UV in neuroblastoma cells, and the neuroprotective effect might be exerted through interfering with p38 and JNK pathways. Moreover, RBM3 exerts its cell-protective effects by modulating PERK-eIF2α-CHOP signaling (Zhou et al., 2017). The PERK-eIF2α-CHOP signaling pathway is one of three main branches involved in unfolded protein response (UPR) activation, and it is involved in UPR-induced apoptosis (Hetz, 2012). The effects of RBM3 on UPR-induced apoptosis have been studied. The research uncovered a hypothermia induced RBM3 expression, and that RBM3 represses the phosphorylation of PERK and eIF2α. CHOP expression was downregulated by phosphorylation of PERK and eIF2α, and EIF2α phosphorylation and CHOP protein expression were elevated in human embryonic kidney HEK293 cells by specific small interfering RNAs and in hippocampal organotypic slice cultured from RBM3 knockout mice (Zhu et al., 2016b). In summary, Cirbp and RBM3 have neuroprotective effects in nerve injury and may provide a potential therapeutic target for the neuroprotection.

## Conclusion

Hypothermia therapy has been proven neuroprotective in the patients suffering from neural injuries such as cardiac arrest and stroke, as established by many studies on the subject. However, the mechanistic aspects are not clearly understood. Clearly, more work is needed, including determination of best strategies to induce hypothermia, improving the protection and clarifying the mechanism. As discussed in this article, local hypothermia may be the best option for providing protection similar to general hypothermia and for reducing temperature effects throughout the body with minimal side effects. Several neuroprotective strategies are being tested to enhance hypothermia protection. Further, cold induced proteins are important in hypothermia protection. The discussion here should provide guidance for future animal studies and clinical trials on hypothermia neuroprotection.

## Author Contributions

Y-JS and Z-YZ prepared first draft of the manuscript. All authors edited the review article. G-YL approved the submission of the manuscript. All authors contributed to the writing, editing, and agree to the submission of the manuscript.

## Conflict of Interest Statement

The authors declare that the research was conducted in the absence of any commercial or financial relationships that could be construed as a potential conflict of interest.
